# Impact of Drying Process on Grindability and Physicochemical Properties of Celery

**DOI:** 10.3390/foods13162585

**Published:** 2024-08-18

**Authors:** Stanisław Rudy, Dariusz Dziki, Beata Biernacka, Renata Polak, Andrzej Krzykowski, Anna Krajewska, Renata Stanisławczyk, Mariusz Rudy, Jagoda Żurek, Grzegorz Rudzki

**Affiliations:** 1 Department of Thermal Technology and Food Process Engineering, University of Life Sciences in Lublin, Głęboka St. 31, 20-612 Lublin, Poland; stanislaw.rudy@up.lublin.pl (S.R.); dariusz.dziki@up.lublin.pl (D.D.); beata.biernacka@up.lublin.pl (B.B.); renata.polak@up.lublin.pl (R.P.); andrzej.krzykowski@up.lublin.pl (A.K.); 2 Department of Agricultural Processing and Commodity Science, Institute of Food and Nutrition Technology, College of Natural Sciences, University of Rzeszow, Zelwerowicza St. 4, 35-601 Rzeszów, Poland; rstanislawczyk@ur.edu.pl (R.S.); mrudy@ur.edu.pl (M.R.); 3 Department of Financial Markets and Public Finance, Institute of Economics and Finance, College of Social Sciences, University of Rzeszow, Ćwiklińskiej 2, 35-601 Rzeszów, Poland; jzurek@ur.edu.pl; 4 Department of Endocrinology, Diabetology, and Metabolic Diseases, Medical University of Lublin, Jaczewski St. 8, 20-090 Lublin, Poland; grzegorz.rudzki@umlub.pl

**Keywords:** celery, freeze drying, vacuum drying, convection drying, microwave drying, drying kinetics, antioxidant activity

## Abstract

The objective of this study was to evaluate the impact of various drying methods: freeze drying, vacuum drying, convection drying, and convection-microwave drying at microwave powers of 50 W and 100 W, along with process temperatures (40 °C, 60 °C, and 80 °C), on the drying kinetics, selected physicochemical properties of dried celery stems, and their grindability. The Page model was employed to mathematically describe the drying kinetics across the entire measurement range. Convection-microwave drying significantly reduced the drying time compared to the other methods. The longest drying duration was observed with freeze drying at 40 °C. The product obtained through freeze drying at 40 °C exhibited the least alteration in color coordinates, the highest antioxidant capacity, and the greatest retention of chlorophylls and total carotenoids. At a specific temperature, the quality of the product obtained from vacuum drying was slightly lower compared to that from freeze drying. The most substantial changes in the physicochemical properties of the dried product were observed with convection-microwave drying at a microwave power of 100 W. The drying method selected had a significant impact on the energy consumption of grinding, average particle size, and the grinding energy index of the dried celery stems; these parameters worsened as the drying temperature increased. The product with the best quality characteristics and disintegration parameters was achieved using freeze drying at 40 °C.

## 1. Introduction

Celery (*Apium graveolens* L.), a member of the *Apiaceae* family, originates from the Mediterranean region. It encompasses three cultivated taxonomic varieties: stalk celery (var. *dulce*), root celery (var. *rapaceum*), and leaf celery (var. *secalinum*) [1,2,3]. Today, celery stalks are grown and consumed globally [4]. Celery is utilized in the food, cosmetic, medical, and chemical industries due to its unique sweet-spicy flavor and its rich content of fiber, carotene, protein, vitamins A and C, minerals, carboxylic acids, amino acids, and phenolic compounds [2,5]. Its antioxidant properties are attributed to its biologically active compounds. The distinctive taste and aroma of celery are primarily due to essential oils, including terpenes, aldehydes, and phthalides [6,7]. Additionally, celery serves as a herbal raw material, providing benefits such as lowering blood pressure, preventing cancer [8], and treating endocrine disorders and infertility [9]. The high water content in celery stalks contributes to their short shelf life and susceptibility to microbial growth. Consequently, employing an effective drying method that preserves the maximum amount of its bioactive substances is essential for extending its shelf life [10].

During the process of moisture removal from dried materials, various physicochemical transformations occur. The alterations in physicochemical properties observed during drying are influenced by both external and internal factors. External factors, which vary according to the drying method used, primarily include temperature, the quantity and type of energy supplied, air flow rate and humidity, the pressure under which the drying process is conducted, and the rate of freezing. Internal factors include the initial moisture content, the manner in which water is bound within the raw material, the glass transition temperature, the thickness of the drying material layer, and its characteristic dimensions [11,12,13].

Convective drying is widely employed in the preservation of fruits and vegetables because of its cost-effectiveness, although it necessitates elevated temperatures to enhance drying efficiency [10,14]. More than 85% of dried food items are processed using diverse convective drying systems, selected primarily for their accessibility, straightforward design, and operational simplicity [15,16]. This approach leads to notable reductions in bioactive compounds due to prolonged processing times and exposure to oxygen [17,18,19]. Consequently, alternative drying methods are implemented to counteract the detrimental effects of convective drying on the quality of dried products. An example of such a method involves convective microwave drying. Microwave radiation heats the dried material volumetrically, enhancing water diffusion rates and resulting in a considerable decrease in drying duration [20,21]. The use of microwave radiation significantly influences drying kinetics, particularly during the falling rate phase [22,23]. Concurrent air circulation helps mitigate excessive overheating of the material, thereby preventing burning, a concern especially pronounced when microwave radiation alone is used during drying [24,25]. This can lead to the degradation of the physicochemical properties of the final dried product [26,27].

Vacuum drying takes place in a low-pressure environment, facilitating the evaporation of water from the material’s surface at a reduced temperature [28]. The pressure within the drying chamber remains above the triple point pressure of water and generally does not surpass 30 kPa [29,30]. The primary factor governing the kinetics of the drying process is the pressure difference between the surface of the material undergoing drying and the condenser that extracts water vapor from the apparatus. The energy necessary for the process is provided through direct contact or by employing microwave or infrared radiation [31,32].

Sublimation drying encompasses several stages: first, freezing the raw material, followed by sublimating the frozen ice, and finally, vacuum drying the remaining unfrozen water within the material. Successful execution of sublimation drying requires maintaining chamber pressure and material temperature below the triple point of water [33,34]. This method is particularly favored for drying heat-sensitive food products due to its operation under high vacuum conditions and low temperatures [35,36].

Research on the impact of drying conditions and methods on specific physicochemical attributes of celery powder is scarce. Previous investigations commonly involved comparing powder parameters obtained at different process temperatures. This study aimed to explore how temperatures during sublimation, vacuum, convective, and convective microwave drying affect the process kinetics and key quality traits of celery stem powder.

## 2. Materials and Methods

### 2.1. Material

The experimental material comprised celery stalks of the Imperial variety, obtained from a local producer near Lublin. The inedible portions of the celery were excised, and the stalks were cut into slices approximately 5 mm thick. An average sample of the raw material was then prepared. Irrespective of the drying technique employed, the material was dried to reach a targeted final moisture content of 10%.

### 2.2. Drying Method

The sublimation and vacuum drying processes were conducted using the ALPHA 1-4 freeze dryer from Martin Christ, which uses a contact heating method. This apparatus includes a drying chamber, a heating plate power system, a water vapor freezing system, and a control and measurement system with an interface. The dryer features a WPT 5 scale integrated with a computer, facilitating continuous monitoring of the drying material’s mass. Sublimation drying was carried out at temperatures of 40 °C, 60 °C, and 80 °C with a chamber pressure of 52 Pa, whereas vacuum drying was performed at the same temperatures with a chamber pressure of 2000 Pa. The raw material was arranged in a single layer (5 mm thick) on the plates. Each experiment was repeated five times.

The convective and convective microwave drying processes were performed using a PROMIS-TECH dryer. Air flowed through the bottom of the dish, perpendicular to the drying material layer, at a speed of 0.5 m/s, measured below the dryer’s sieve. The material and dish were placed on a Radwag laboratory scale with an accuracy of 0.1 g. During mass measurements, the power supply to the dish-rotating motor and the airflow were turned off. Mass was recorded every five minutes. Convective drying was conducted at temperatures of 40 °C, 60 °C, and 80 °C. For convective microwave drying, the same temperatures were used, along with microwave powers of 50 W and 100 W (power per 100 g of raw material). Temperature stabilization was maintained by a heater and a temperature probe situated behind the fan and before the airflow speed control system. The device allowed for smooth adjustment of microwave power (at a frequency of 2450 MHz), setting the drying temperature, and controlling the airflow speed. An integrated computer program enabled the monitoring of current operating parameters and the export of data to a spreadsheet. The drying process was repeated five times until a final moisture content of 10% was achieved.

### 2.3. Modeling of Drying Curves

Irrespective of the drying method examined, the water content (absolute humidity) in the dried celery at each measurement point was calculated using the following equation:

(1) $$u_{\tau }=\frac{m-m_{s}}{m_{s}}$$

where

m—mass of the material at a specific measurement point [g],

m_s_—dry matter content in celery [g].

The final sample mass (m_k_), at which it achieves the desired final relative humidity, was computed using the following expression:

(2) $$m_{k}=m_{p}\cdot \frac{100-w_{p}}{100-w_{k}}$$

where

m_p_—initial mass [g],

w_p_—initial moisture content of the raw material [%],

w_k_—final desired relative humidity of the dried product [%].

The drying kinetics were expressed by the variation of reduced water content (MR) over drying time:

(3) $$MR=\frac{u_{\tau }-u_{r}}{u_{p}-u_{r}}$$

where

u_τ_—the water content at a specific measurement point [kg·kg_s.s_^−1^],

u_p_—the initial water content [kg·kg_s.s_^−1^],

u_r_—the equilibrium water content [kg·kg_s.s_^−1^].

The equilibrium water content after sublimation, vacuum, and convective microwave drying is very low; thus, it was assumed that the equilibrium moisture content (u_r_) is 0 across the entire measurement range.

Six of the most commonly used models from the literature were employed to describe the drying curves for sublimation, vacuum, convective, and convective-microwave methods. The equations of these models are presented in Table 1.

The equilibrium water content following sublimation, vacuum, and convective microwave drying is exceedingly low, leading to the assumption that the equilibrium moisture content (u_r_) is 0 throughout the entire measurement range. Six widely cited models from the literature were utilized to characterize the drying curves of sublimation, vacuum, convective, and convective-microwave methods. The equations of these models are compiled in Table 1.

### 2.4. Color Measurement

Color measurement was conducted using a reflective method with an X-Rite 8200 spherical spectrophotometer equipped with a 12.7 mm measurement aperture. The D65 light source and a standard 10° colorimetric observer were utilized. Prior to each measurement, the instrument underwent calibration using a white reference standard. Color measurements were carried out in five replicates for the finely ground (<100 µm) dried sample.

Color coordinates were determined within the CIELab* color space. In this system, color characterization involves the numerical representation of three coordinates: L*, a*, and b*. Here, L* signifies brightness, ranging from 0 for a perfectly black object to 100 for a perfectly white one. Coordinate a* denotes color variation from green (−a*) to red (+a*), while b* indicates variation from blue (−b*) to yellow (+b*).

Based on the obtained color coordinates, values for chroma (c) and hue angle (h) of the dried sample were computed in cylindrical coordinates [43]:

(4) $$c=\sqrt{{\left(a^{*}\right)}^{2}+{\left(b^{*}\right)}^{2}}$$

(5) $${h=\tan}^{-1}\frac{b^{*}}{a^{*}}$$

The browning index (BI) was calculated based on the following relationship: [44]:

(6) $$BI=\frac{\left[100\left(x-0.31\right)\right]}{0.17}$$

The trichromatic coordinate x (as defined in Equation (10)) was derived through the transformation of the CIELab color space to the CIEXYZ color space, employing the following equations for this purpose [44]:

(7) $$X=X_{n}{\left(\frac{a^{*}}{500}+\frac{L^{*}+16}{116}\right)}^{3}$$

(8) $$Y=Y_{n}{\left(\frac{L^{*}+16}{116}\right)}^{3}$$

(9) $$Z=Z_{n}{\left(\frac{{-b}^{*}}{200}+\frac{L^{*}+16}{116}\right)}^{3}$$

(10) $$x=\frac{X}{X+Y+Z}$$

where

X, Y, Z—color coordinates in the CIEXYZ system,

X_n_ = 94.81, Y_n_ = 100, Z_n_ = 107.3—parameters of the reference white point,

X—trichromatic coordinate in the XYY color system.

### 2.5. Antiradical Activity

In this study, the capacity to counteract ABTS (2,2′-azinobis (3-ethylbenzothiazoline-6-sulfonic acid)) radicals followed the methodology developed by Re et al. [45], while the capability to counteract DPPH (2,2-diphenyl-1-picrylhydrazyl) radicals adhered to the protocol outlined by Brand-Williams et al. [46]. Absorbance reduction was measured quantitatively using a spectrophotometer at wavelengths of 734 nm for ABTS and 517 nm for DPPH. The effectiveness of neutralizing ABTS and DPPH radicals was quantified as the EC50 value, representing the dry mass concentration (mg∙mL^−1^) required to achieve a 50% decrease in the initial concentration of either ABTS or DPPH radicals.

### 2.6. The Content of Total Carotenoids and Chlorophylls

Chlorophyll a, chlorophyll b, and total carotenoid content were determined using spectrophotometric methods with a Diode Array absorbance spectrophotometer, Hewlett-Packard 8453, operating in the wavelength range of 190–1100 nm. The assay procedure followed the method of Lichtenthaler [47]. This method involves pigment extraction using an 80% acetone solution, followed by absorbance measurements at wavelengths specific to chlorophyll a, chlorophyll b, and carotenoids.

A measured amount of sample was transferred to a porcelain mortar and ground with 3 mL of 80% aqueous acetone for 2 min. The resulting suspension was centrifuged at 15,000× *g* (g = 9.81 m/s^2^) for 3 min, and the supernatant was quantitatively collected and its volume measured. Subsequently, 100 μL of the supernatant was pipetted and mixed with 2 mL of 80% aqueous acetone for absorbance spectrum measurements at wavelengths characteristic of chlorophyll a, chlorophyll b, and carotenoids, specifically 470 nm, 646.8 nm, and 663.2 nm, respectively. Quartz cuvettes from Sigma were used for absorbance spectrum measurements.

The content of chlorophyll a, chlorophyll b, and total carotenoids was calculated using the following formulas:

(11) $$C_{a}=12.25\cdot A_{663.2}-2.79\cdot A_{646.8}$$

(12) $$C_{a}=21.50\cdot A_{646.8}-5.10\cdot A_{663.2}$$

(13) $$C_{x+c}=\frac{1000\cdot A_{470}-1.82\cdot C_{a}-85.02\cdot C_{b}}{198}$$

### 2.7. Fragmentation of the Dried Material

The disintegration process of the dried material was carried out using a GRINDOMIX GM 200 knife mill, produced by Retsch. The grinder’s operational elements included two stainless steel blades, each 1 mm thick, arranged on opposite sides of the shaft at varying heights. A 100-g sample of the dried material was placed into the device’s grinding chamber. The grinding operation was executed at a shaft rotation speed of 7000 rpm, lasting for 30 s. To mitigate the influence of the final moisture content on the grinding process, the samples were preconditioned in a climate chamber at 20 °C and 50% relative humidity for 48 h before disintegration. Measurements were taken in five replicates. The detailed procedure and measurement setup were described by Dziki et al. [48].

#### 2.7.1. Granulometric Composition of the Dried Material and the Average Particle Size

The crushed, dried material underwent granulometric composition analysis using a Retsch AS 200 vibratory sieve shaker. The apparatus was equipped with a series of sieves with mesh sizes of 800 µm, 600 µm, 400 µm, 200 µm, and 100 µm. A 20-g sample of the dried material was sieved for 2 min at a vibration amplitude of 1.5 mm. Each fraction was then weighed, and its percentage composition was calculated. The measurements were repeated five times. The average particle size (ds) was determined based on the granulometric composition using the appropriate formula [49]:

(14) $$d_{s}=\frac{\sum_{i=1}^{i=u}h_{i}\cdot P_{i}}{100}$$

where

h_i_—the mean value of the class interval,

P_i_—the percentage contribution of the given class,

u—the number of sieves used.

#### 2.7.2. Energy Consumption Indices of Grinding

The specific energy consumption of grinding was determined by calculating the energy used for grinding per kilogram of dried material. The experiments were conducted in five replicates. The grinding efficiency index for dried celery was calculated as the ratio of the surface area generated by grinding to the energy consumed for the grinding process. These indices were determined according to the methodology described in [48].

### 2.8. Statistical Analysis

A single-factor analysis of variance was conducted. Tukey’s test was used to assess the significance of differences between the means. All experiments and analyses were conducted in five replicates. Nonlinear regression analysis of the drying kinetics was performed using the least squares method, determining the coefficient of determination, root mean square error (RMSE), and reduced chi-square test values (χ^2^). The RMSE and χ^2^ values were derived from the following relationships:

(15) $$RMSE=\sqrt{\frac{\sum_{i=1}^{N}{\left({MR}_{i,p}-{MR}_{i,e}\right)}^{2}}{N}}$$

(16) $$\chi^{2}=\frac{\sum_{i=1}^{N}{\left({MR}_{i,p}-{MR}_{i,e}\right)}^{2}}{N-n}$$

where

MR_i,p_—predicted value of reduced moisture content,

MR_i,e_—experimental value of reduced moisture content,

N—number of measurements,

n—number of parameters in the equation of the model. 

Statistical analysis was conducted using Statistica 13 software by StatSoft. All calculations were performed assuming a significance level of α = 0.05.

## 3. Results and Discussion

### 3.1. Drying Kinetics

**Figure 1:**
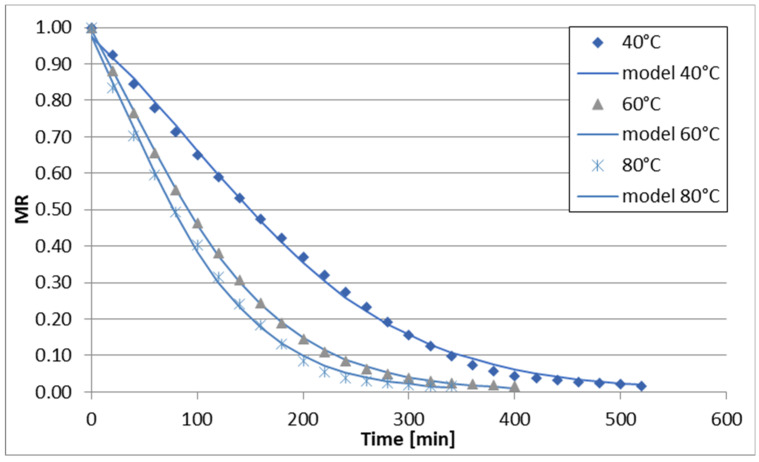
Drying curves of the freeze-drying process.

**Figure 2:**
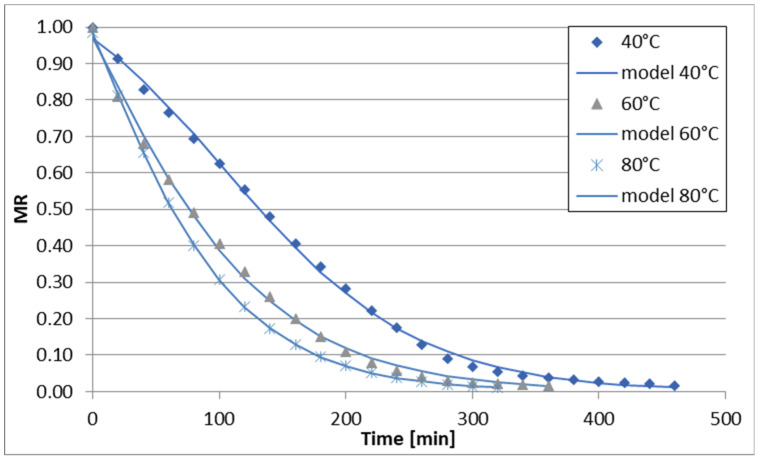
Drying curves of the vacuum-drying process.

**Figure 3:**
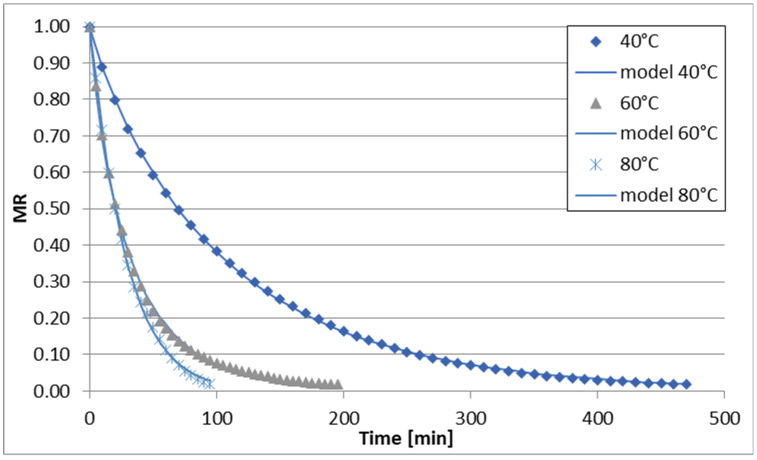
Drying curves of the air-drying process.

**Figure 4:**
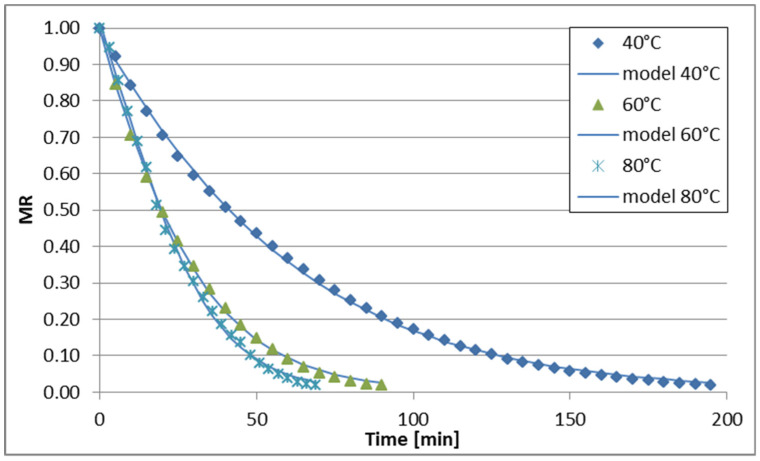
Drying curves of the microwave-air drying process (50 W).

**Figure 5:**
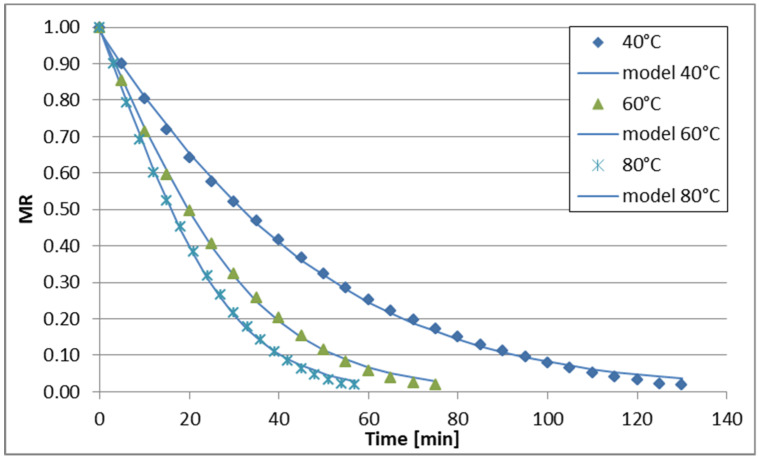
Drying curves of the microwave-air drying process (100 W).

The changes in reduced moisture content (MR) over time during sublimation, vacuum, convective, and convective microwave drying (at two microwave power levels) of celery stalks are illustrated in Figure 1, Figure 2, Figure 3, Figure 4 and Figure 5. Across all drying methods studied, the duration of the drying process decreased as the temperature increased. Sublimation drying exhibited the longest drying times at each temperature level, while convective microwave drying at 100 W microwave power showed the shortest times. For instance, at 40 °C, sublimation drying lasted 520 min, whereas convective microwave drying at 80 °C with 100 W microwave power required the shortest time. Increasing the temperature from 60 °C to 80 °C (during sublimation and vacuum drying) slightly reduced drying times by 60 min and 40 min, respectively. Convective drying of celery stalks at 80 °C was approximately 80% shorter than at 40 °C and 59% shorter than at 60 °C. Convective-microwave drying at 50 W and 60 °C took approximately 46% of the time required at 20 °C, and approximately 35% of the time at 80 °C. Increasing the microwave power from 50 W to 100 W reduced drying times by approximately 33.3% (at 40 °C), 16.7% (at 60 °C), and 17.4% (at 80 °C).

The regression analysis results for the six models examined to describe the kinetics of sublimation, vacuum, convection, and convection-microwave drying are summarized in Table 2, Table 3, Table 4, Table 5 and Table 6. It is evident that for each of the analyzed models (with the exception of the Wang and Singh model for convection drying at 60 °C, for which the coefficient of determination (R2) was 0.7948), the experimental data were well-fitted. The coefficient of determination for the equations ranged from 0.9181 to 0.9999 across the entire measurement range. Additionally, the root mean square error (RMSE) and the reduced chi-square (χ2) values were low, falling within the ranges of 0.0079–0.0726 and 0.0001–0.0043, respectively. Depending on the drying method and conditions, the best fit to the experimental data was obtained with the Page and logistic models. However, the application of the logistic model to convection drying at 40 °C and 60 °C did not yield statistically significant results for describing the changes in reduced water content over drying time. Therefore, the Page model was utilized for all drying methods (Figure 1, Figure 2, Figure 3, Figure 4 and Figure 5). During the drying process of fruits and vegetables, the Page model frequently provided the best fit to the experimental data, regardless of the drying method used [37,50,51,52].

The coefficients for the six regression equations analyzed are detailed in Table 7 (sublimation drying), Table 8 (vacuum drying), Table 9 (convection drying), and Table 10 and Table 11 (convection microwave drying).

An examination of the effect of drying methods on the *k* coefficient reveals that, within a given model, the coefficient is lowest during freeze drying, slightly higher during vacuum drying, and considerably higher during convective and convective microwave drying, with the AMD100 method showing the highest values. The variation in the drying coefficient k is directly linked to the degree of change in the physicochemical properties of the dried product, where a rise in *k* is associated with a decline in product quality.

### 3.2. Color Assessment

Across most of the measurement range (excluding the AMD100 sample at 80 °C), the L* color coordinate of dried celery stalks exceeded that of the raw material (Table 12, Figure S1). As the temperature increased from 40 °C to 80 °C, the L* value declined for products dried by sublimation, vacuum, and convection methods. Products obtained via two convection-microwave methods exhibited the highest brightness at 60 °C and the lowest at 80 °C. When comparing all drying methods, the sublimation-dried product exhibited the highest brightness at each temperature level, followed by vacuum, convection, and convection-microwave drying at 50 W (except at 60 °C, where the convection-microwave dried product was brighter than the convection-dried product). The lowest brightness across all analyzed temperatures was observed in the convection microwave-dried product at 100 W. The L* values for sublimation and vacuum-dried products showed minimal differences, especially at the two lower temperatures. The products dried using AD, AMD50, and AMD100 methods were notably darker. The smallest changes in L* value with increasing temperature from 40 °C to 80 °C were observed in sublimation-dried products (approximately 3.6 units), while the largest changes occurred with the AMD50 method (approximately 9.2 units).

The raw material exhibited the lowest color saturation (c) value. The color saturation for sublimation and vacuum-dried products was similar, with no significant differences in four instances. For the other three drying methods, color intensity decreased with increasing temperature. Convection-microwave dried products (at both microwave powers) showed higher c values at 40 °C and 80 °C and lower at 60 °C. The influence of microwave power on c was significant only at 40 °C; at higher temperatures, microwave power did not affect color saturation. The lowest c value was noted in the convection-dried product at 80 °C, and the highest in the sublimation-dried product at 40 °C. Similar to color saturation, the hue value (h) of dried celery stalks was minimally affected by the temperature in sublimation and vacuum drying, with only slightly lower values for the FD80 method. The hue value was significantly lower for convection and convection microwave-dried products (50 W and 100 W). As the drying agent temperature increased, the h value of dried celery stalks decreased. An exception was seen in convection drying at 40 °C and 60 °C, where hue differences were negligible. At each temperature level, convection-dried products had higher h values than convection microwave-dried products. Increasing microwave power reduced the h value at each drying agent temperature.

The lowest browning index (BI) was found in fresh celery stalks. BI values for vacuum-dried products were independent of drying temperature (within the analyzed range). For sublimation drying, BI values at each temperature were slightly higher than those for vacuum drying, though these differences were small and often statistically insignificant. For the other three drying methods, BI values were higher than for sublimation and vacuum-dried products, except for AD60, where values were comparable. Among AD, AMD50, and AMD100 methods, the lowest BI was observed at 60 °C, higher at 40 °C, and highest at 80 °C. Microwave-assisted convection drying increased the BI compared to convection drying at the same temperature. Higher microwave power further increased the BI at each temperature level.

The changes in color parameters analyzed in this study primarily depend on the drying method and temperature, consistent with findings by Kręcisz et al. in celery stalk drying [9]. Other researchers agree that sublimation drying results in the brightest and least browning-susceptible products [53,54]. It is generally accepted that replacing convection drying with microwave or convection-microwave drying yields a product with a color closer to the raw material [55,56,57]. However, this method’s disadvantages are uneven heating, overheating, and cavitation, especially at higher microwave powers [58,59]. These phenomena can lead to surface scorching and unfavorable color changes [60]. The changes in color coordinates observed in this study corroborate these assertions.

### 3.3. Antioxidant Activity

The drying process of celery stalks reduces the antioxidant capacity of the dried product against both tested radicals, regardless of the drying method and conditions, in comparison to the antioxidant capacity of the raw material (Table 13, Figure S2). For all examined drying methods and conditions, the EC_50_ coefficient was higher for the DPPH radical than for ABTS*. As the temperature increased, the EC_50_ value (for both ABTS* and DPPH) increased in the dried celery stalks across all drying methods. At each temperature level, the highest antioxidant potential against both radicals was observed for sublimation drying, followed closely by vacuum drying. Dalamau et al. [61] and Anatal et al. [62] reported significantly higher reductions in antioxidant capacity after sublimation drying. Products obtained via convection and convection-microwave methods exhibited significantly lower antioxidant potential compared to those dried using FD and VD methods, with the most pronounced differences observed at higher temperatures (60 °C and 80 °C). The use of convection-microwave drying instead of convection drying decreased the EC50 value (enhanced the antioxidant potential of the dried product). Increasing microwave power negatively affected the antioxidant capacity against both analyzed radicals. The lowest antioxidant capacities were observed in products dried by the AD method at 80 °C for ABTS* and AMD100 at 60 °C for DPPH, with antioxidant potentials approximately three times lower than those of the raw material. Rout et al. [63] found significantly higher antioxidant potentials in Indian borage leaves after sublimation and vacuum drying compared to convection drying, aligning with the results of this study. However, differing results were obtained for microwave drying, where the antioxidant potential was comparable to sublimation drying and independent of microwave power. Additionally, as temperature increased, antioxidant potential also increased [63]. These relationships may be due to greater stresses at higher temperatures accelerating the damage to plant tissues, resulting in the release of more active compounds [64]. Moreover, new active compounds, such as organic acids, furans, and alcohols, may form at high temperatures [65].

### 3.4. Total Carotenoids and Chlorophylls Content

Each drying method analyzed resulted in a reduction in total carotenoid, chlorophyll a, and chlorophyll b content compared to their levels in the raw celery stalks (Table 14, Figure S3). The sublimation drying process consistently yielded the highest levels of chlorophyll a and b, as well as total carotenoids, with retention rates ranging from 81.5% to 93.2% for carotenoids, 78% to 98.7% for chlorophyll a, and 70% to 94.7% for chlorophyll b across temperatures from 40 °C to 80 °C. Relative to the raw material, sublimation-dried products consistently exhibited lower levels of total carotenoids and chlorophylls at each temperature, with minimal differences at lower temperatures within the range studied, but more significant differences observed at a hot plate temperature of 80 °C. Vacuum drying resulted in approximately 1.7% lower retention of carotenoids, 5.5% lower for chlorophyll a, and 5.4% lower for chlorophyll b compared to sublimation drying at this temperature. Both convection and convection-microwave drying methods showed a decrease in the content of these compounds as the drying temperature increased. Convection-microwave drying at 50 W demonstrated the highest total carotenoid content at each temperature level, followed by conventional convection drying, while the lowest content was observed in convection-microwave drying at 100 W. The relationship for chlorophyll a and b content was less consistent, with microwave radiation showing a positive effect on celery stalk preservation, albeit an increase in microwave power generally resulted in decreased chlorophyll content. Ahmed et al. [66] reported that higher air drying temperatures for coriander leaves led to greater losses of chlorophyll and total carotenoids, with a threefold decrease in total carotenoid content observed as temperatures increased from 45 °C to 65 °C. In a similar study, Feng et al. [67] compared chlorophyll content in dried lettuce cubes using four methods, finding the highest retention in sublimation-dried products, slightly lower retention in tray-dried products with microwave-assisted heating, and the lowest chlorophyll content in conventionally dried products [67].

### 3.5. The Susceptibility of the Dried Product to Fragmentation

In most instances, the temperature of heating plates (sublimation and vacuum drying) and the drying air temperature (convection and convection-microwave drying) had a significant impact on the particle size distribution of dried celery stem powder (Table 15). Across all drying methods, as temperatures rose, there was a noticeable shift in the particle size distribution towards larger fractions. Specifically, celery stem powder obtained from sublimation drying showed the highest proportion of the two smallest fractions (<100 µm, 100–200 µm) at each temperature level, constituting approximately 90% at 40 °C, 85% at 60 °C, and 80% at 80 °C. Moreover, sublimation-dried powder exhibited slightly lower quantities of the finest particle fractions, with the combined content of the two smallest fractions totaling approximately 81% at 40 °C, 74% at 60 °C, and 69% at 80 °C. Conversely, celery stem powder from convection drying exhibited a markedly higher presence of larger fractions, with the predominant fraction being in the 400–600 µm size range, accounting for approximately 22–28% across the entire temperature spectrum.

In the case of convection-microwave drying, an increase in microwave power resulted in a shift of the powder’s particle size distribution towards coarser fractions, regardless of the drying air temperature. At each microwave power level, elevating the air-drying temperature led to an increase in the proportion of larger fractions and a decrease in the proportion of smaller fractions. The finest particle size distribution of convection microwave-dried powder was achieved at an air-drying temperature of 40 °C with 50 W microwave power, while the coarsest powder was obtained at 80 °C with 100 W microwave power. At all temperature levels, the particle size distribution of convection microwave-dried powder exhibited finer particles compared to that from convection drying (Table 15).

Fante and Norena [54] investigated the impact of blanching, sublimation, and convection drying (50–70 °C) on the average particle size of garlic after comminution. They found that the average particle size of sublimation-dried powder was approximately half of that of convection-dried powder at 70 °C. Additionally, an increase in air drying temperature led to a reduction in average particle size. The authors attributed the smaller particle size after sublimation drying to greater porosity, while after convection drying, it was due to drying to equilibrium moisture content. Jung et al. [68] provided a broader perspective on the influence of moisture content on powder susceptibility to comminution. They observed similar effects of the drying method on particle size distribution in dried mealworms [69]. Eliasson et al. [70] explored the effects of temperature and drying methods (convection, convection microwave, and sublimation) on the comminution efficiency of compressed blackcurrant powder. According to their findings, temperature during convection and convection-microwave drying minimally affected particle size distribution. The fraction sized 710–1250 µm was predominant for both drying methods. Furthermore, finer powder was obtained from convection drying compared to convection-microwave drying. Sublimation drying resulted in more than double the number of fractions smaller than 500 µm compared to both convection and convection-microwave drying. The authors attributed the increased susceptibility to comminution in sublimation-dried powder to better material structure retention and higher porosity [70].

Irrespective of the drying method employed, the specific energy consumption for grinding increased as the heating plate temperature (air drying temperature) rose. The sublimation drying method exhibited the lowest specific energy consumption for grinding (E_jr_), whereas convection drying consistently showed the highest Ejr across all analyzed temperature levels. The impact of drying air temperature on specific energy consumption for grinding was most pronounced with convection drying. Sublimation drying yielded the lowest E_jr_ at a heating plate temperature of 20 °C, whereas convection drying yielded the highest E_jr_ at 80 °C (Table 16). Specific energy consumption values for grinding ranged from 83.2 kJ·kg^−1^ to 162.1 kJ·kg^−1^, significantly higher compared to the energy required for grinding pear pomace, which ranged from 8.83 to 9.07 kJ·kg^−1^ for sublimation drying and from 12.06 to 12.66 kJ·kg^−1^ for contact drying [71].

Across all drying conditions analyzed, the average particle size (d_s_) ranged from 151.9 µm to 447.6 µm. Sublimation drying consistently produced the smallest mean particle diameters, followed slightly larger by vacuum drying (particle diameter increase ranging from 23% to 36%). The mean particle size of celery stem powder obtained from convection drying was approximately two and a half times larger than that obtained from sublimation drying at comparable temperatures. Convection-microwave drying reduced d_s_ across all analyzed temperatures and microwave powers compared to convection drying, resulting in d_s_ reductions ranging from 0.3% to 18%, depending on process conditions. Decreasing microwave power (convection-microwave drying) led to smaller mean particle sizes, ds, across the three analyzed air-drying temperatures. For instance, reducing microwave power from 100 W to 50 W decreased mean particle sizes by 36.3 µm at 40 °C, 52.8 µm at 60 °C, and 70.2 µm at 80 °C (Table 16).

## 4. Conclusions

Across all examined drying methods, the Page model provided the best fit to the experimental data depicting the change in reduced moisture content over drying time. Convective microwave drying at 100 W microwave power emerged as the most advantageous method in terms of drying duration, whereas sublimation drying appeared the least favorable. Sublimation-dried celery, particularly at temperatures of 40 °C and 60 °C, exhibited minimal changes in the analyzed color coordinates, the highest antioxidant capacity, and the greatest retention of chlorophylls and total carotenoids, albeit vacuum-dried celery showed slightly inferior results. The choice of drying method significantly impacts the energy consumption of grinding, average particle size, and grinding energy index of celery stalks. As temperature increased, all evaluated quality characteristics of the dried celery and indices reflecting the level of disintegration worsened across all drying methods. Consequently, sublimation or vacuum drying methods prove most advantageous based on these criteria, with convective drying yielding the least favorable outcomes.

## Figures and Tables

**Table 1:** Mathematical models for drying curve analysis.

Number	Model Name	Equation
1	Newton [37]	MR=exp⁡(−k·τ)
2	Page [38]	$MR=exp(-k\cdot \tau^{n})$
3	Henderson i Pabis [39]	MR=a⋅exp⁡(−k·τ)
4	Logarithmic [40]	$MR=a\cdot \exp\left(-k\cdot \tau \right)+b$
5	Wang i Singh [41]	$MR=1+a\cdot \tau +b{\cdot \tau }^{2}$
6	Logistic [42]	$MR=\exp\left(-k\cdot \tau^{n}\right)+b\cdot \tau$

k—drying coefficient (min^−1^); a, b—coefficients of the equations; n—exponent; τ—time (min).

**Table 2:** Statistical analysis of models describing kinetics of freeze drying of celery stems.

Model	Temperature								
	40 °C			60 °C			80 °C		
	RMSE	χ^2^	R^2^	RMSE	χ^2^	R^2^	RMSE	χ^2^	R^2^
Newton	0.0510	0.0012	0.9729	0.0355	0.0004	0.9865	0.0345	0.0004	0.9873
Page	0.0187	0.0002	0.9964	0.0079	0.0001	0.9993	0.0170	0.0001	0.9969
Henderson and Pabis	0.044	0.0009	0.9798	0.0299	0.0003	0.9904	0.0317	0.0003	0.9892
Logarithmic	0.0222	0.0002	0.9949	0.0204	0.0002	0.9955	0.0201	0.0001	0.9957
Wang i Singh	0.0076	0.0001	0.9994	0.0178	0.0001	0.9966	0.0174	0.0001	0.9967
Logistic	0.0125	0.0001	0.9984	0.0044	0.0001	0.9998	0.0128	0.0001	0.9983

**Table 3:** Statistical analysis of models describing kinetics of vacuum drying of celery stems.

Model	Temperature								
	40 °C			60 °C			80 °C		
	RMSE	χ^2^	R^2^	RMSE	χ^2^	R^2^	RMSE	χ^2^	R^2^
Newton	0.0607	0.0015	0.9638	0.0246	0.0002	0.9930	0.0216	0.0001	0.9947
Page	0.0203	0.0002	0.9960	0.0181	0.0001	0.9962	0.0126	0.0001	0.9982
Henderson and Pabis	0.0527	0.0011	0.9727	0.024	0.0002	0.9933	0.0204	0.0001	0.9953
Logarithmic	0.0341	0.0005	0.9886	0.0154	0.0001	0.9972	0.0157	0.0001	0.9972
Wang i Singh	0.0200	0.0002	0.9961	0.0300	0.0003	0.9896	0.0355	0.0004	0.9857
Logistic	0.0124	0.0001	0.9985	0.0144	0.0001	0.9976	0.0099	0.0001	0.9989

**Table 4:** Statistical analysis of models describing kinetics of air drying of celery stems.

Model	Temperature								
	40 °C			60 °C			80 °C		
	RMSE	χ^2^	R^2^	RMSE	χ^2^	R^2^	RMSE	χ^2^	R^2^
Newton	0.0143	0.0002	0.9968	0.0230	0.0004	0.9908	0.0117	0.0001	0.9984
Page	0.0029	0.0001	0.9999	0.0092	0.0001	0.9985	0.0071	0.0001	0.9994
Henderson and Pabis	0.0096	0.0001	0.9986	0.0201	0.0003	0.9930	0.0101	0.0001	0.9988
Logarithmic	0.0080	0.0001	0.9990	0.0094	0.0001	0.9985	0.0054	0.0001	0.9996
Wang i Singh	0.0726	0.0043	0.9181	0.1087	0.0079	0.7948	0.0376	0.0005	0.9829
Logistic	-	-	-	-	-	-	0.0073	0.0001	0.9994

**Table 5:** Statistical analysis of models describing kinetics of air microwave drying of celery stems (50 W).

Model	Temperature								
	40 °C			60 °C			80 °C		
	RMSE	χ^2^	R^2^	RMSE	χ^2^	R^2^	RMSE	χ^2^	R^2^
Newton	0.0114	0.0001	0.9983	0.0160	0.0001	0.9970	0.0445	0.0008	0.9794
Page	0.0088	0.0001	0.9990	0.0084	0.0001	0.9992	0.0100	0.0001	0.9990
Henderson and Pabis	0.0110	0.0001	0.9984	0.0145	0.0001	0.9975	0.0341	0.0005	0.9879
Logarithmic	0.0042	0.0001	0.9998	0.0043	0.0001	0.9998	0.0172	0.0001	0.9969
Wang i Singh	0.0366	0.0009	0.9823	0.0311	0.0003	0.9886	0.0156	0.0001	0.9975
Logistic	0.0074	0.0001	0.9993	0.0070	0.0001	0.9994	0.0113	0.0001	0.9987

**Table 6:** Statistical analysis of models describing kinetics of air microwave drying of celery stems (100 W).

Model	Temperature								
	40 °C			60 °C			80 °C		
	RMSE	χ^2^	R^2^	RMSE	χ^2^	R^2^	RMSE	χ^2^	R^2^
Newton	0.0178	0.0001	0.9961	0.0305	0.0003	0.9900	0.0421	0.0005	0.9848
Page	0.0108	0.0001	0.9986	0.0116	0.0001	0.9986	0.0131	0.0001	0.9985
Henderson and Pabis	0.0163	0.0001	0.9967	0.0269	0.0002	0.9922	0.0356	0.0004	0.9891
Logarithmic	0.0025	0.0001	0.9999	0.0084	0.0001	0.9992	0.0125	0.0001	0.9987
Wang i Singh	0.0270	0.0003	0.9901	0.0142	0.0001	0.9978	0.0084	0.0001	0.9994
Logistic	0.0090	0.0001	0.9990	0.0090	0.0001	0.9991	0.0098	0.0001	0.9992

**Table 7:** Coefficient values in the models describing the freeze drying of celery stems.

Temperature	Equation	Coefficient			
		a	k	n	b
40 °C	Newton		0.005453		
	Page		0.000866	1.341947	
	Henderson and Pabis	1.079352	0.005863		
	Logarithmic	1.199114	0.004105		−0.167331
	Wang and Singh	−0.003990			0.000004
	Logistic	1.341249	0.010007		0.376062
60 °C	Newton		0.008639		
	Page		0.002459	1.254195	
	Henderson and Pabis	1.061544	0.009134		
	Logarithmic	1.100403	0.007774		−0.062254
	Wang and Singh	−0.006145			0.000009
	Logistic	1.659136	0.013630		0.668080
80 °C	Newton		0.010162		
	Page		0.003586	1.216927	
	Henderson and Pabis	1.042217	0.010559		
	Logarithmic	1.089738	0.008764		−0.072520
	Wang and Singh	−0.007188			0.000013
	Logistic	1.649052	0.015640		0.684655

**Table 8:** Coefficient values in the models describing the vacuum drying of celery stems.

Temperature	Equation	Coefficient			
		a	k	n	b
40 °C	Newton		0.006380		
	Page		0.000637	1.441252	
	Henderson and Pabis	1.092733	0.006925		
	Logarithmic	1.206207	0.004937		−0.158260
	Wang and Singh	−0.004668			0.000006
	Logistic	1.216830	0.013164		0.252796
60 °C	Newton		0.009925		
	Page		0.005667	1.115811	
	Henderson and Pabis	1.017217	0.010087		
	Logarithmic	1.049855	0.008725		−0.052833
	Wang and Singh	−0.006933			0.000012
	Logistic	2.131982	0.013255		1.190890
80 °C	Newton		0.011981		
	Page		0.006612	1.128017	
	Henderson and Pabis	1.023179	0.012242		
	Logarithmic	1.042993	0.011159		−0.032541
	Wang and Singh	−0.008184			0.000017
	Logistic	2.274244	0.015901		1.306220

**Table 9:** Coefficient values in the models describing the air drying of celery stems.

Temperature	Equation	Coefficient			
		a	k	n	b
40 °C	Newton		0.009387		
	Page		0.014580	0.910181	
	Henderson and Pabis	0.959386	0.008993		
	Logarithmic	0.955972	0.009390		0.011777
	Wang and Singh	−0.005976			0.000009
	Logistic				
60 °C	Newton		0.030171		
	Page		0.052991	0.848384	
	Henderson and Pabis	0.951945	0.028601		
	Logarithmic	0.947198	0.032376		0.029727
	Wang and Singh	−0.016011			0.000061
	Logistic				
80 °C	Newton		0.035453		
	Page		0.028675	1.060004	
	Henderson and Pabis	1.018408	0.036099		
	Logarithmic	1.033477	0.033537		−0.025982
	Wang and Singh	−0.025310			0.000164
	Logistic	5.229394	0.039952		4.215170

**Table 10:** Coefficient values in the models describing the air microwave drying of celery stems (50 W).

Temperature	Equation	Coefficient			
		a	k	n	b
40 °C	Newton		0.017427		
	Page		0.014255	1.047227	
	Henderson and Pabis	1.010552	0.017610		
	Logarithmic	1.027051	0.016040		−0.032235
	Wang and Singh	−0.012455			0.000040
	Logistic	4.152973	0.019920		3.210918
60 °C	Newton		0.037046		
	Page		0.026993	1.090498	
	Henderson and Pabis	1.021095	0.037807		
	Logarithmic	1.047784	0.033581		−0.043481
	Wang and Singh	−0.026477			0.000179
	Logistic	3.092393	0.045273		2.114567
80 °C	Newton		0.040419		
	Page		0.014795	1.299569	
	Henderson and Pabis	1.087218	0.043823		
	Logarithmic	1.173050	0.033330		−0.123665
	Wang and Singh	−0.029875			0.000229
	Logistic	1.678319	0.065736		0.655823

**Table 11:** Coefficient values in the models describing the air microwave drying of celery stems (100 W).

Temperature	Equation	Coefficient			
		a	k	n	b
40 °C	Newton		0.023128		
	Page		0.016128	1.091323	
	Henderson and Pabis	1.022819	0.023656		
	Logarithmic	1.061554	0.020158		−0.062139
	Wang and Singh	−0.017049			0.000076
	Logistic	2.854433	0.028733		1.889380
60 °C	Newton		0.038969		
	Page		0.020221	1.191710	
	Henderson and Pabis	1.042204	0.040555		
	Logarithmic	1.114797	0.031977		−0.101687
	Wang and Singh	−0.028595			0.000211
	Logistic	1.896838	0.056679		0.913850
80 °C	Newton		0.049167		
	Page		0.022588	1.245014	
	Henderson and Pabis	1.059706	0.052012		
	Logarithmic	1.152914	0.038972		−0.130020
	Wang and Singh	−0.036268			0.000339
	Logistic	1.668718	0.077356		0.682626

**Table 12:** Influence of drying method and temperature on the color of celery stalks.

Sample	L^*^	c	h	BI
RM	62.1 ± 0.63 ^m^	16.7 ± 0.42 ^h^	102.4 ± 1.72 ^i^	25.07 ± 0.574 ^a^
FD 40 °C	84.9 ± 0.59 ^l^	30.8 ± 0.28 ^g^	110.3 ± 0.84 ^gh^	30.20 ± 1.021 ^bc^
FD 60 °C	83.3 ± 0.58 ^k^	29.5 ± 0.45 ^f^	110.0 ± 0.40 ^fg^	29.58 ± 0.669 ^b^
FD 80 °C	81.3 ± 0.44 ^j^	29.5 ± 0.24 ^f^	108.5 ± 0.67 ^f^	31.64 ± 0.484 ^cd^
VD 40 °C	82.0 ± 0.41 ^j^	29.2 ± 0.32 ^f^	110.5 ± 0.66 ^gh^	29.44 ± 0.773 ^b^
VD 60 °C	80.0 ± 0.47 ^i^	29.0 ± 0.56 ^f^	111.8 ± 0.97 ^h^	29.02 ± 1.224 ^b^
VD 80 °C	77.1 ± 0.50 ^h^	27.6 ± 0.22 ^e^	110.5 ± 0.40 ^gh^	29.54 ± 0.381 ^b^
AD 40 °C	71.3 ± 0.49 ^f^	24.9 ± 0.23 ^c^	104.2 ± 0.56 ^e^	33.30 ± 0.432 ^de^
AD 60 °C	69.6 ± 0.51 ^e^	22.7 ± 0.77 ^b^	104.2 ± 0.5 ^e^	30.59 ± 1.305 ^bc^
AD 80 °C	68.8 ± 0.32 ^e^	21.0 ± 0.40 ^a^	91.0 ± 0.97 ^b^	35.15 ± 0.779 ^e^
AMD50 40 °C	69.2 ± 0.55 ^e^	26.6 ± 0.43 ^d^	99.4 ± 0.38 ^d^	41.27 ± 1.066 ^g^
AMD50 60 °C	72.9 ± 0.48 ^g^	22.4 ± 0.55 ^b^	96.6 ± 0.73 ^c^	32.66 ± 1.077 ^d^
AMD50 80 °C	63.7 ± 0.44 ^b^	22.2 ± 0.31 ^b^	90.8 ± 0.63 ^b^	41.34 ± 0.203 ^gh^
AMD100 40 °C	65.2 ± 0.54 ^c^	25.1 ± 0.39 ^c^	96.5 ± 0.38 ^c^	43.34 ± 0.73 ^hi^
AMD100 60 °C	67.4 ± 0.75 ^d^	22.2 ± 0.44 ^b^	92.3 ± 0.17 ^b^	37.86 ± 1.349 ^f^
AMD100 80 °C	61.1 ± 0.61 ^a^	22.1 ± 0.34 ^b^	88.2 ± 1.03 ^a^	44.41 ± 1.213 ^i^

RM—raw material, FD 40 °C—freeze drying 40 °C, FD 60 °C—freeze drying 60 °C, FD 80 °C—freeze drying 80 °C, VD 40 °C—vacuum drying 40 °C, VD 60 °C—vacuum drying 60 °C, VD 80 °C—vacuum drying 80 °C, AD 40 °C—air (convective) drying 40 °C, AD 60 °C—air (convective) drying 60 °C, AD 80 °C—air (convective) drying 80 °C, AMD50 40 °C—air (convective) drying 40 °C with 50 W microwaves, AMD50 60 °C—air (convective) drying 60 °C with 50 W microwaves, AMD50 80 °C—air (convective) drying 80 °C with 50 W microwaves, AMD100 40 °C—air (convective) drying 40 °C with 100 W microwaves, AMD100 60 °C—air (convective) drying 60 °C with 100 W microwaves, AMD100 80 °C—air (convective) drying 80 °C with 100 W microwaves, L*—lightness, c—chroma, h—hue angle, BI—browning index; the values are expressed as mean ± SD; means with different letter superscript are significantly different (α = 0.05).

**Table 13:** The antioxidant capacity of dried celery stalks.

Sample	ABTS (EC_50_; mg DM·mL^−1^)	DPPH (EC_50_; mg DM·mL^−1^)
RM	31.5 ± 0.42 ^a^	54.4 ± 0.72 ^a^
FD 40 °C	40.2 ± 0.85 ^c^	61.6 ± 0.95 ^b^
FD 60 °C	45.2 ± 0.69 ^c^	77.6 ± 0.90 ^c^
FD 80 °C	52.4 ± 0.97 ^d^	108.3 ± 1.26 ^d^
VD 40 °C	51.1 ± 0.57 ^d^	123.8 ± 1.36 ^e^
VD 60 °C	52.3 ± 0.68 ^de^	126.5 ± 1.31 ^g^
VD 80 °C	53.0 ± 0.97 ^e^	131.0 ± 1.65 ^h^
AD 40 °C	58.2 ± 0.80 ^h^	127.5 ± 1.28 ^g^
AD 60 °C	62.2 ± 0.58 ^g^	135.5 ± 1.55 ^i^
AD 80 °C	100.0 ± 0.55 ^j^	152.3 ± 1.64 ^j^
AMD50 40 °C	55.8 ± 0.74 ^f^	113.8 ± 1.28 ^f^
AMD50 60 °C	60.7 ± 0.75 ^j^	135.0 ± 1.54 ^i^
AMD50 80 °C	69.6 ± 0.69 ^k^	178.9 ± 1.02 ^l^
AMD100 40 °C	57.9 ± 0.88 ^i^	157.7 ± 1.65 ^k^
AMD100 60 °C	61.4 ± 0.85 ^j^	211.4 ± 1.60 ^n^
AMD100 80 °C	76.0 ± 0.56 ^k^	203.8 ± 1.48 ^m^

RM—raw material, FD 40 °C—freeze drying 40 °C, FD 60 °C—freeze drying 60 °C, FD 80 °C—freeze drying 80 °C, VD 40 °C—vacuum drying 40 °C, VD 60 °C—vacuum drying 60 °C, VD 80 °C—vacuum drying 80 °C, AD 40 °C—air (convective) drying 40 °C, AD 60 °C—air (convective) drying 60 °C, AD 80 °C—air (convective) drying 80 °C, AMD50 40 °C—air (convective) drying 40 °C with 50 W microwaves, AMD50 60 °C—air (convective) drying 60 °C with 50 W microwaves, AMD50 80 °C—air (convective) drying 80 °C with 50 W microwaves, AMD100 40 °C—air (convective) drying 40 °C with 100 W microwaves, AMD100 60 °C—air (convective) drying 60 °C with 100 W microwaves, AMD100 80 °C—air (convective) drying 80 °C with 100 W microwaves; the values are expressed as mean ± SD; means with different letter superscript are significantly different (α = 0.05).

**Table 14:** The total carotenoid and chlorophyll content in dried celery stalks.

Sample	Carotenoids (mg·100 g_d.m_^−1^)	Chlorophyll a (mg·100 g_d.m_^−1^)	Chlorophyll b (mg·100 g_d.m_^−1^)
RM	102.7 ± 0.77 ^k^	461.5 ± 3.01 ^l^	140.2 ± 0.85 ^l^
FD 40 °C	95.7 ± 1.09 ^j^	455.5 ± 4.23 ^k^	132.8 ± 1.37 ^k^
FD 60 °C	87.9 ± 10 ^i^	412.5 ± 2.21 ^j^	117.8 ± 1.83 ^j^
FD 80 °C	83.7 ± 1.28 ^gh^	360.1 ± 3.71 ^h^	98.1 ± 1.29 ^f^
VD 40 °C	95.1 ± 0.20 ^j^	450.4 ± 3.08 ^k^	132.5 ± 3.10 ^k^
VD 60 °C	84.8 ± 0.59 ^h^	401.2 ± 1.36 ^i^	108.4 ± 1.55 ^i^
VD 80 °C	82.0 ± 0.99 ^g^	334.5 ± 1.3 ^g^	90.6 ± 0.80 ^g^
AD 40 °C	71.5 ± 0.41 ^d^	293.8 ± 2.42 ^d^	84.7 ± 1.10 ^f^
AD 60 °C	58.3 ± 1.41 ^b^	250.5 ± 2.87 ^b^	77.4 ± 2.18 ^d^
AD 80 °C	57.9 ± 0.90 ^b^	250.1 ± 1.84 ^b^	62.0 ± 1.26 ^a^
AMD50 40 °C	77.7 ± 0.21 ^f^	319.8 ± 2.31 ^f^	82.9 ± 2.72 ^ef^
AMD50 60 °C	73.7 ± 1.20 ^e^	270.2 ± 2.93 ^c^	78.0 ± 1.26 ^d^
AMD50 80 °C	70.5 ± 0.65 ^d^	250.1 ± 1.93 ^b^	70.2 ± 0.84 ^c^
AMD100 40 °C	60.9 ± 0.59 ^c^	300.5 ± 3.06 ^e^	82.1 ± 1.08 ^ef^
AMD100 60 °C	53.4 ± 1.03 ^a^	273.0 ± 1.83 ^c^	80.0 ± 1.52 ^de^
AMD100 80 °C	51.9 ± 1.46 ^a^	233.1 ± 2.24 ^a^	65.9 ± 1.29 ^b^

RM—raw material, FD 40 °C—freeze drying 40 °C, FD 60 °C—freeze drying 60 °C, FD 80 °C—freeze drying 80 °C, VD 40 °C—vacuum drying 40 °C, VD 60 °C—vacuum drying 60 °C, VD 80 °C—vacuum drying 80 °C, AD 40 °C—air (convective) drying 40 °C, AD 60 °C—air (convective) drying 60 °C, AD 80 °C—air (convective) drying 80 °C, AMD50 40 °C—air (convective) drying 40 °C with 50 W microwaves, AMD50 60 °C—air (convective) drying 60 °C with 50 W microwaves, AMD50 80 °C—air (convective) drying 80 °C with 50 W microwaves, AMD100 40 °C—air (convective) drying 40 °C with 100 W microwaves, AMD100 60 °C—air (convective) drying 60 °C with 100 W microwaves, AMD100 80 °C—air (convective) drying 80 °C with 100 W microwaves; the values are expressed as mean ± SD; means with different letter superscript are significantly different (α = 0.05).

**Table 15:** Particle size distribution of celery powder (%).

Sample	Fractions (µm)					
	>800	800–600	600–400	400–200	200–100	<100
FD 40 °C	0.43 ± 0.358 ^a^	1.06 ± 0.463 ^a^	1.96 ± 0.600 ^a^	6.58 ± 0.479 ^a^	36.15 ± 0.228 ^i^	53.82 ± 0.340 ^k^
FD 60 °C	1.05 ± 0.461 ^ab^	1.31 ± 0.267 ^a^	2.36 ± 0.351 ^a^	10.02 ± 0.189 ^c^	34.57 ± 0.186 ^h^	50.69 ± 0.360 ^j^
FD 80 °C	1.73 ± 0.301 ^bc^	1.64 ± 0.560 ^a^	3.26 ± 0.316 ^b^	13.25 ± 0.456 ^e^	37.45 ± 0.23 ^j^	42.67 ± 0.318 ^i^
VD 40 °C	1.92 ± 0.399 ^c^	2.85 ± 0.746 ^b^	3.76 ± 0.346 ^b^	10.72 ± 0.456 ^c^	39.65 ± 0.455 ^k^	41.10 ± 0.460 ^h^
VD 60 °C	4.29 ± 0.376 ^d^	3.86 ± 0.449 ^b^	5.75 ± 0.181 ^c^	11.81 ± 0.334 ^d^	35.60 ± 0.310 ^i^	38.69 ± 0.509 ^g^
VD 80 °C	4.32 ± 0.483 ^d^	3.57 ± 0.536 ^b^	5.16 ± 0.323 ^c^	17.63 ± 0.410 ^g^	38.45 ± 0.477 ^j^	30.87 ± 0.261 ^f^
AD 40 °C	10.26 ± 0.28 ^h^	11.32 ± 0.671 ^c^	22.36 ± 0.191 ^g^	23.80 ± 0.423 ^j^	19.02 ± 0.330 ^d^	13.24 ± 0.493 ^b^
AD 60 °C	13.58 ± 0.25 ^i^	12.80 ± 0.694 ^d^	26.47 ± 0.279 ^h^	18.62 ± 0.413 ^h^	15.60 ± 0.496 ^b^	12.93 ± 0.349 ^ab^
AD 80 °C	14.57 ± 0.272 ^j^	14.38 ± 0.211 ^fg^	27.98 ± 0.472 ^i^	20.53 ± 0.216 ^i^	10.29 ± 0.588 ^a^	12.25 ± 0.227 ^a^
AMD50 40 °C	6.24 ± 0.535 ^e^	10.67 ± 0.151 ^c^	15.32 ± 0.393 ^d^	16.26 ± 0.285 ^f^	28.37 ± 0.193 ^g^	23.14 ± 0.451 ^e^
AMD50 60 °C	7.28 ± 0.471 ^f^	13.72 ± 0.582 ^ef^	15.30 ± 0.392 ^d^	18.18 ± 0.082 ^gh^	25.11 ± 0.330 ^f^	20.41 ± 0.468 ^d^
AMD50 80 °C	10.35 ± 0.615 ^h^	15.95 ± 0.208 ^h^	17.17 ± 0.549 ^e^	14.12 ± 0.375 ^e^	24.78 ± 0.419 ^f^	17.63 ± 0.328 ^c^
AMD100 40 °C	7.82 ± 0.215 ^f^	13.48 ± 0.329 ^de^	18.48 ± 0.446 ^f^	15.54 ± 0.228 ^f^	24.51 ± 0.402 ^f^	20.17 ± 0.190 ^d^
AMD100 60 °C	8.89 ± 0.225 ^g^	15.60 ± 0.286 ^gh^	28.06 ± 0.289 ^i^	10.24 ± 0.551 ^c^	20.36 ± 0.551 ^e^	16.85 ± 0.617 ^c^
AMD100 80 °C	13.14 ± 0.103 ^i^	17.65 ± 0.344 ^i^	30.18 ± 0.252 ^j^	8.34 ± 0.460 ^b^	17.42 ± 0.488 ^c^	13.27 ± 0.363 ^b^

RM—raw material, FD 40 °C—freeze drying 40 °C, FD 60 °C—freeze drying 60 °C, FD 80 °C—freeze drying 80 °C, VD 40 °C—vacuum drying 40 °C, VD 60 °C—vacuum drying 60 °C, VD 80 °C—vacuum drying 80 °C, AD 40 °C—air (convective) drying 40 °C, AD 60 °C—air (convective) drying 60 °C, AD 80 °C—air (convective) drying 80 °C, AMD50 40 °C—air (convective) drying 40 °C with 50 W microwaves, AMD50 60 °C—air (convective) drying 60 °C with 50 W microwaves, AMD50 80 °C—air (convective) drying 80 °C with 50 W microwaves, AMD100 40 °C—air (convective) drying 40 °C with 100 W microwaves, AMD100 60 °C—air (convective) drying 60 °C with 100 W microwaves, AMD100 80 °C—air (convective) drying 80 °C with 100 W microwaves; the values are expressed as mean ± SD; means with different letter superscript are significantly different (α = 0.05).

**Table 16:** Specific grinding energy, average final particle size, and grinding efficiency index from dried celery stalks.

Sample	E_jr_ (kJ·kg^−1^)	d_s_ (μm)	W_er_ (m^2^·MJ^−1^)
FD 40 °C	83.2 ± 1.29 ^a^	151.9 ± 3.41 ^a^	0.627 ± 0.010 ^k^
FD 60 °C	86.9 ± 1.09 ^c^	160.8 ± 4.27 ^a^	0.583 ± 0.010 ^j^
FD 80 °C	98.9 ± 0.74 ^d^	181.6 ± 3.72 ^b^	0.451 ± 0.005 ^g^
VD 40 °C	83.9 ± 1.34 ^ab^	187.2 ± 7.46 ^b^	0.506 ± 0.006 ^i^
VD 60 °C	86.0 ± 1.66 ^bc^	219.2 ± 3.21 ^c^	0.483 ± 0.007 ^h^
VD 80 °C	102.9 ± 0.92 ^e^	226.9 ± 1.78 ^c^	0.349 ± 0.005 ^f^
AD 40 °C	137.0 ± 0.87 ^k^	385.6 ± 5.63 ^f^	0.192 ± 0.003 ^b^
AD 60 °C	146.0 ± 0.65 ^l^	421.0 ± 3.65 ^h^	0.175 ± 0.003 ^a^
AD 80 °C	162.1 ± 1.13 ^m^	447.6 ± 5.96 ^i^	0.163 ± 0.002 ^a^
AMD50 40 °C	118.8 ± 0.68 ^g^	314.9 ± 4.78 ^d^	0.264 ± 0.004 ^e^
AMD50 60 °C	124.5 ± 0.89 ^h^	343.8 ± 6.07 ^e^	0.240 ± 0.005 ^d^
AMD50 80 °C	131.3 ± 1.33 ^j^	375.9 ± 3.69 ^f^	0.206 ± 0.004 ^c^
AMD100 40 °C	108.8 ± 0.85 ^f^	351.2 ± 2.63 ^e^	0.271 ± 0.002 ^e^
AMD100 60 °C	110.6 ± 0.60 ^f^	396.6 ± 5.03 ^g^	0.243 ± 0.006 ^d^
AMD100 80 °C	126.9 ± 1.14 ^i^	446.1 ± 2.51 ^i^	0.192 ± 0.004 ^b^

E_jr_—specific energy consumption for grinding, d_s_—average particle size, W_er_—grinding efficiency index, FD 40 °C—freeze drying 40 °C, FD 60 °C—freeze drying 60 °C, FD 80 °C—freeze drying 80 °C, VD 40 °C—vacuum drying 40 °C, VD 60 °C—vacuum drying 60 °C, VD 80 °C—vacuum drying 80 °C, AD 40 °C—air (convective) drying 40 °C, AD 60 °C—air (convective) drying 60 °C, AD 80 °C—air (convective) drying 80 °C, AMD50 40 °C—air (convective) drying 40 °C with 50 W microwaves, AMD50 60 °C—air (convective) drying 60 °C with 50 W microwaves, AMD50 80 °C—air (convective) drying 80 °C with 50 W microwaves, AMD100 40 °C—air (convective) drying 40 °C with 100 W microwaves, AMD100 60 °C—air (convective) drying 60 °C with 100 W microwaves, AMD100 80 °C—air (convective) drying 80 °C with 100 W microwaves; the values are expressed as mean ± SD; means with different letter superscript are significantly different (α = 0.05).

## Data Availability

The original contributions presented in the study are included in the article and Supplementary Material, further inquiries can be directed to the corresponding author.

## Supplementary Materials

The following supporting information can be downloaded at: https://www.mdpi.com/article/10.3390/foods13162585/s1, Figure S1: Influence of drying method and temperature on the color celery stalks; Figure S2: The antioxidant capacity of dried celery stalks; Figure S3: The total carotenoid and chlorophyll content in dried celery stalks.
