# Supplementary material for: Impact of Drying Process on Grindability and Physicochemical Properties of Celery
Source: Foods. 2024 Aug 18;13(16):2585. doi: 10.3390/foods13162585 (PMC11354119; doi:10.3390/foods13162585)
Supplement: Supplementary file 1 [file foods-13-02585-s001.zip › foods-3135414-supplementary.pdf]

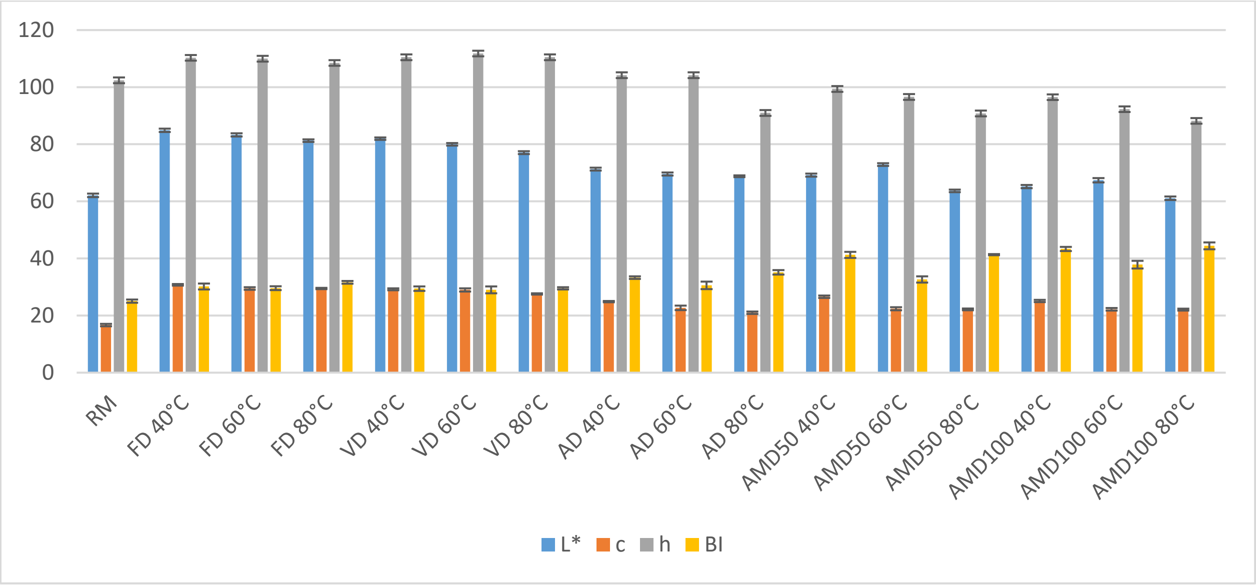

Figure S1. Influence of drying method and temperature on the color celery stalks

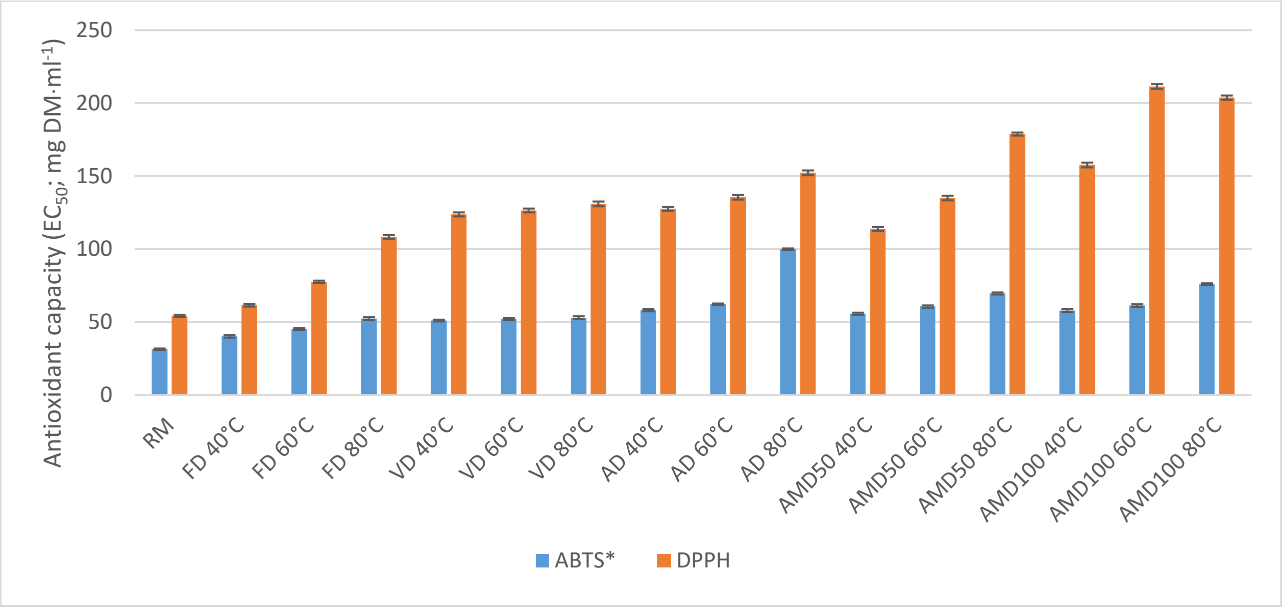

Figure S2. The antioxidant capacity of dried celery stalks

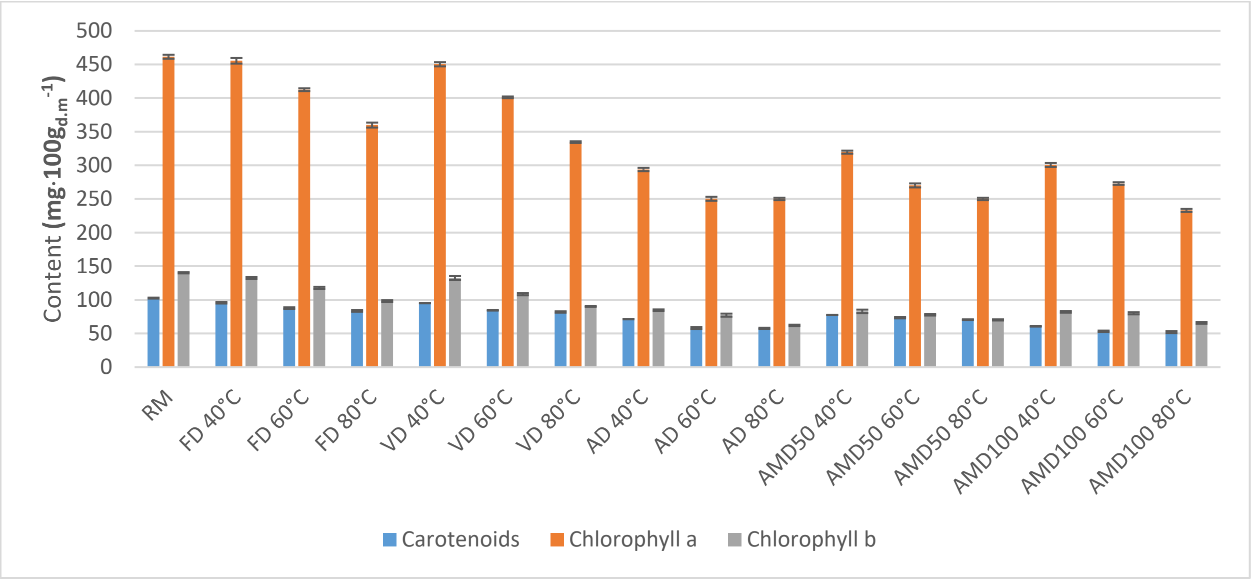

Figure S3. The total carotenoid and chlorophyll content in dried celery stalks
